# The complete chloroplast genome sequence of the fragrant plant *Lavandula angustifolia* (Lamiaceae)

**DOI:** 10.1080/23802359.2018.1431067

**Published:** 2018-01-24

**Authors:** Lan Ma

**Affiliations:** School of Biological and Environmental Engineering, Xi’an University, Xi’an, China

**Keywords:** *Lavandula angustifolia*, Illumina sequencing, chloroplast genome, MITObim

## Abstract

Chloroplast (cp) genome sequences have become a useful popular tool for population and phylogeny recently. It is little known about genetics information of *Lavandula angustifolia*. Here, the complete chloroplast genome of the *L. angustifolia* has been reconstructed from the whole-genome Illumina sequencing data. The circular genome is 153,448 bp in size, and comprises of a pair of inverted repeat (IR) regions of 25,632 bp each, a large single-copy (LSC) region of 84,588 bp and a small single-copy (SSC) region of 17,596 bp. The total GC content is 38.0%, while the corresponding values of the LSC, SSC, and IR regions are 36.2, 31.8, and 43.2%, respectively. The chloroplast genome contains 132 genes, including 88 protein-coding genes, 8 ribosomal RNA genes, and 37 transfer RNA genes. The Maximum-Likelihood phylogenetic analysis showed a strong sister relationship with *Salvia miltiorrhiza* in Lamiaceae. These findings provide a foundation for further investigation of cp genome evolution in *L. angustifolia* and other higher plants.

Many plants in Lamiaceae are used as spices for their fragrance, such as mint, basil, and rosemary. Lavender (*Lavandula angustifolia*, Lamiaceae), native to the Mediterranean, is another common aromatic plant. The oil extracted from lavender flowers with special flavor often added in detergent to make laundry smell good. Most research of lavender focused on their biochemical composition and pharmacological properties (Danh et al. 2013; Angelo et al. 2014; Giovannini et al. 2016), however, it is a rare report on molecular genetics and genome analysis of lavender. Here, we assembled the complete chloroplast genome *L. angustifolia,* which will provide the basis for further studies on its phylogenetics, molecular breeding, and genetic engineering.

The genomic DNA was extracted from *L. angustifolia* that was collected from Qinling Mountains (Shaanxi Province) and stored in our lab. The whole genome shotgun sequencing of *L. angustifolia* was sequenced using the Illumina HiSeq 2000 platform (Illumina, Hayward, CA, USA). Total 20.9 M 125 bp raw reads were retrieved and trimmed by CLC Genomics Workbench v8.0 (CLC Bio, Aarhus, Denmark). A subset of 19.6 M trimmed reads were used for reconstructing the chloroplast genome by MITObim v1.8 (DSM Nutritional Products Ltd, Kaiseraugst, Switzerland) (Hahn et al. 2013), with that of its congener *Perilla setoyensis* (GenBank Accession: NC_030757.1) as the initial reference genome. A total of 1,821,394 individual chloroplast reads yielded an average coverage of 934.6-fold. The chloroplast genome was annotated in GENEIOUS R9 (Biomatters Ltd., Auckland, New Zealand) by aligning with that of *P. setoyensis* (NC_030757.1).

The typical quadripartite chloroplast genome of *L. angustifoli*a (GenBank Accession: NC_029370.1) is a double-stranded circular DNA molecule with 153,448 bp in size. It comprises of a pair of inverted repeat (IR) regions of 25,632 bp each, separated by a large single-copy (LSC) region of 84,588 bp and a small single-copy (SSC) region of 17,596 bp. The total GC content is 38.0%, while the corresponding values of the LSC, SSC, and IR regions are 36.2, 31.8, and 43.2%, respectively.

This chloroplast genome harbors 134 functional genes, including 88 protein-coding genes (PCGs), 37 tRNA genes, and 8 rRNA genes. Six PCGs, seven tRNA genes, and all rRNA genes are duplicated in the IR regions. The LSC region possesses 63 PCGs and 22 tRNA genes, while the SSC region contains 12 PCGs and one tRNA gene. Thirty two PCGs, 17 tRNA genes, and 4 rRNA genes are located in the forward strand while others are located in the reverse strand. Among those genes, 21 are involved in photosynthesis, four in transcription, and two in substance metabolism. Moreover, 12 genes contain one intron, while another two genes harbor two introns; all the other genes are intronless.

The Maximum-Likelihood phylogenetic analysis of *L. angustifolia* using 65 protein-coding genes data among other 10 chloroplast sequences downloaded from the GenBank database (Figure 1). The phylogenetic tree showed the position of *L. angustifolia* was situated as the sister of *Salvia miltiorrhiza* in Lamiaceae. These findings provide a foundation for further investigation of chloroplast genome evolution in *L. angustifolia* and other higher plants.

**Figure 1.**
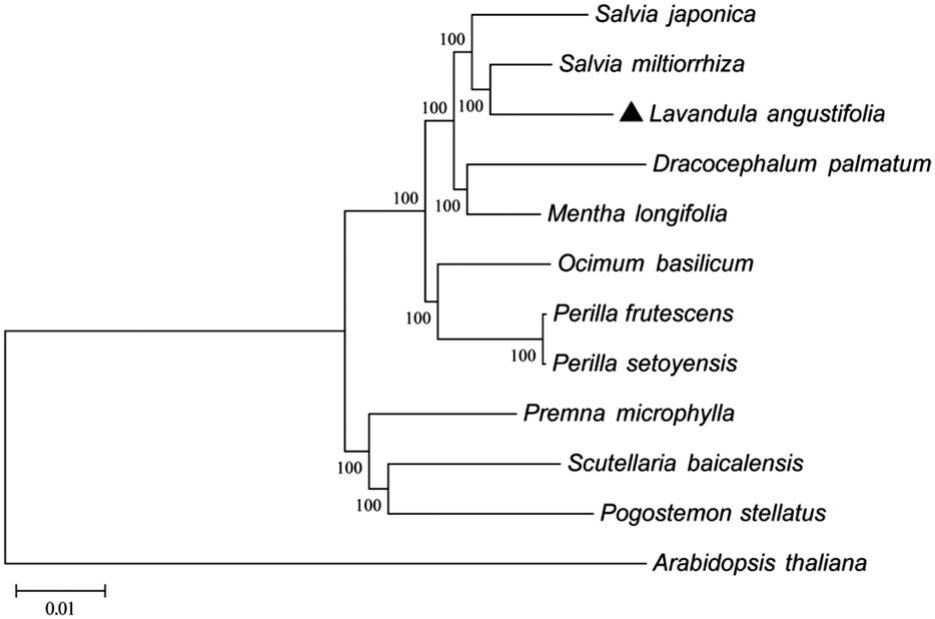
Phylogenetic of 10 species within the family Lamiaceae based on the Maximum-Likelihood analysis of the whole cp genome sequences using 500 bootstrap replicates and setting *Arabidopsis thaliana* (Brassicasaae) as outgroup. The analyzed species and corresponding Genbank accession numbers are as follows: *Arabidopsis thaliana* (NC_000932.1), *Dracocephalum palmatum* (NC_031874.1), *Lavandula angustifolia* (NC_029370.1), *Mentha longifolia* (NC_032054.1), *Ocimum basilicum* (NC_035143.1), *Perilla frutescens* (NC_030756.1), *Perilla setoyensis* (NC_030757.1), *Premna microphylla* (NC_026291.1), *Pogostemon stellatus* (NC_031434.1), *Salvia miltiorrhiza* (NC_020431.1), *Salvia japonica* (NC_035233.1) *Scutellaria baicalensis* (NC_027262.1).
